# Microphone Handling Noise: Measurements of Perceptual Threshold and Effects on Audio Quality

**DOI:** 10.1371/journal.pone.0140256

**Published:** 2015-10-16

**Authors:** Paul Kendrick, Iain R. Jackson, Bruno M. Fazenda, Trevor J. Cox, Francis F. Li

**Affiliations:** Acoustics Research Centre, School of Computing Science and Engineering, University of Salford, Salford, M5 4WT, United Kingdom; University of Kent, UNITED KINGDOM

## Abstract

A psychoacoustic experiment was carried out to test the effects of microphone handling noise on perceived audio quality. Handling noise is a problem affecting both amateurs using their smartphones and cameras, as well as professionals using separate microphones and digital recorders. The noises used for the tests were measured from a variety of devices, including smartphones, laptops and handheld microphones. The signal features that characterise these noises are analysed and presented. The sounds include various types of transient, impact noises created by tapping or knocking devices, as well as more sustained sounds caused by rubbing. During the perceptual tests, listeners auditioned speech podcasts and were asked to rate the degradation of any unwanted sounds they heard. A representative design test methodology was developed that tried to encourage everyday rather than analytical listening. Signal-to-noise ratio (SNR) of the handling noise events was shown to be the best predictor of quality degradation. Other factors such as noise type or background noise in the listening environment did not significantly affect quality ratings. Podcast, microphone type and reproduction equipment were found to be significant but only to a small extent. A model allowing the prediction of degradation from the SNR is presented. The SNR threshold at which 50% of subjects noticed handling noise was found to be 4.2 ± 0.6 dBA. The results from this work are important for the understanding of our perception of impact sound and resonant noises in recordings, and will inform the future development of an automated predictor of quality for handling noise.

## Introduction

Impulsive, transient noises such as door slams, coughs, pops and scratches or noises generated by handling devices are often a problem when recordings. They can occur on most types of recording devices such as portable recorders, mobile phones, smartphones, tablets, cameras or camcorders, with or without separate microphones. Handling noise occurs when the recording device, or something it is attached to, such as a microphone stand or cable, is inadvertently knocked or brushed. A survey into recording errors found that handling noise was a common problem often audible in user generated recordings [1]. The generating mechanisms of the noises can be broadly split into [2]: (i) Mechanical noise caused by vibrations transmitted to the capsule from other components within the device, such as a loose cable connection. (ii) Poorly designed internal shock mounts, which although intended to reduce handling noise exhibit resonant behaviour in the audible region. (iii) The structure onto which the microphone is mounted (e.g. a microphone stand) has a resonant vibration that is transmitted to the capsule.

The aim of this study was to understand the perceptual effects of handling noise on subjective ratings of sound quality and determine its audibility in terms of absolute thresholds. The test methodology was based on the audition of speech podcasts which were corrupted with various types of impact and rubbing noises introduced at various levels. Subjects listened to the podcasts and were asked to detect and rate the level of degradation to the sound quality. As far as the authors are aware, this is the first time the impact of microphone handling noises on audio quality has been investigated experimentally.

## Method

There are currently a number of standards for the evaluation of audio quality [3,4,5] that aim to quantify judgements using methods from psychophysics. Such perceptual procedures frequently place subjects in artificial situations where their listening mode is often influenced by the test method. Truax [6] describes two methods of listening for non-natural environments: analytical and distracted. Subjects asked to undertake perceptual testing on sound quality tend to use analytical listening methods to pick out degradations by comparing the audio to an uncorrupted reference. On the other hand, for the general public listening to audio in their everyday lives, the perception of the degradations might be better thought of as distracted listening. The listener is focussed on the foreground sound in the audio, and the degradations are mostly in the background of perception. Our interest lay in this latter style of listening.

To reduce the biases introduced by analytical listening a representative design [7] was employed where the test methodology best represented ‘the behavioural setting to which the results are intended to apply’ [8]. The question of representative test design is often raised within Soundscape research, where in-situ studies such as sound walks might be used [9]. While the use of a sound walk puts the subject in an environment that is less artificial than a laboratory, such methods are still vulnerable to the listening mode being changed because the subject is knowingly taking part in a scientific study.

The representative design for the microphone handling noise tests involved radio podcasts being presented to listeners over the internet. Allowing the users to perform the test over the internet ensures that the environment and playback equipment is representative of many everyday cases. It also reaches out to a much wider audience than a classical laboratory test, thus assuring a larger and potentially more diverse sample [10]. The subjects were asked to answer questions about the podcast’s content, to focus attention on the foreground speech and to move attention away from the degradations. Participants were asked to report the presence of any sound that degraded the audio quality. If a noise was reported, the subject was then asked to rate the degradation.

In classical trial-by-trial testing, participants are typically presented with one condition/one instance of an error per trial, and asked to rate the quality of each trial. Such a format can lead to the presence of a range of test artefacts, demand characteristics and cognitive biases in the data. Participants quickly learn the format of the test and can adjust expectations and attention accordingly. Large numbers of trials are often required, increasing the risk of participant fatigue. Ratings of quality are even vulnerable to the temporal location of an error within a trial [11]. The current experiment was designed to minimise some of these effects by requiring participants to remain continuously engaged with the task and interrupt the playback themselves on hearing an unwanted noise, preventing the formation of expectations for how often and when errors ought to occur. More details on the experimental design are given in the following sections.

### Generation of handling noise examples

Three exemplar devices were chosen to create the samples for the perceptual tests: a popular dynamic microphone (Shure SM58), a mobile phone (iPhone 4) and a lapel microphone (Audio Technica AT803b). Handling noises can be impulsive, due to impact events, or may be more sustained, when something rubs or brushes against audio equipment. Consequently, examples of handling noises were recorded in an anechoic chamber where the experimenter made a variety of impulsive sounds by tapping for four minutes, and then an additional four minutes of noise examples by rubbing or brushing. The physical manipulations were applied to the microphones, audio devices, supporting stands and connecting cables. The sensitivities of each device were captured by playing a 1 kHz calibration tone over a Genelec 1029a loudspeaker at 1 meter for about 10 seconds, in an anechoic environment. The loudspeaker had previously been calibrated using a sound level meter to produce 84 dB at the same position. The sounds have been catalogued into a database which can be downloaded from S1 Audio.

The noises generated were then processed into three classes: short, medium and long sounds. In order to segment the handling noises into individual events the L_10_ (the level exceed for 10% of the time) was computed. Individual peaks were identified where the level exceeded the L_10_ value. Short sounds were identified as regions with a single peak or a collection of peaks spaced less than 125 ms apart. 125 ms was chosen because it is the fast integration period in sound level meter standards [12]. Longer sounds were identified as contiguous sounds with no gaps between peaks longer than 125 ms, where the length of each group of sounds was computed as the time between the first and last peak. Medium and long sounds were labelled as those below and above the 50% percentile length respectively.

**Fig 1:**
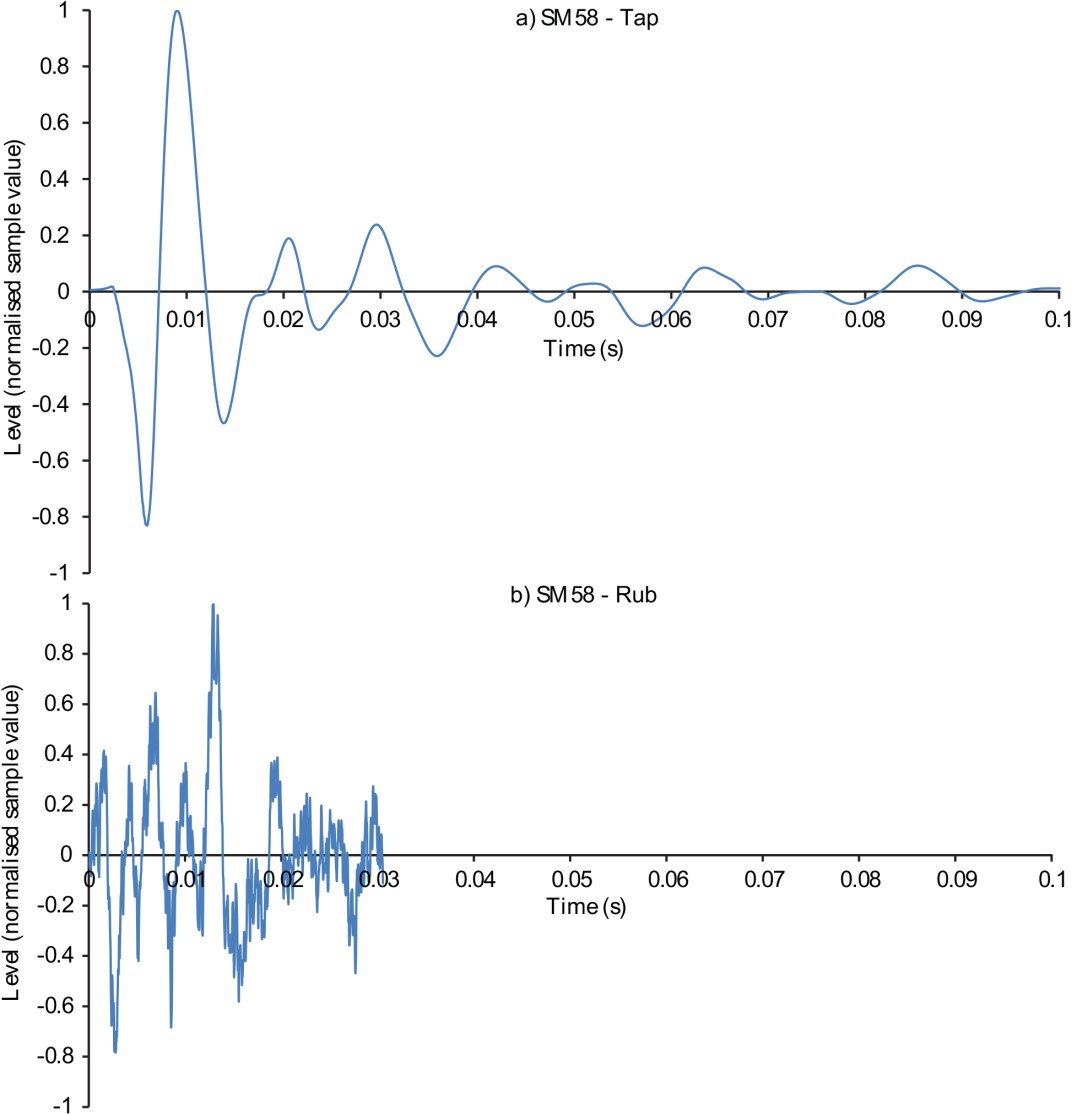
Examples waveforms of microphone handling noises recorded from a dynamic SM58 microphone. a) Tapping and b) rubbing type handling noises.

**Fig 2:**
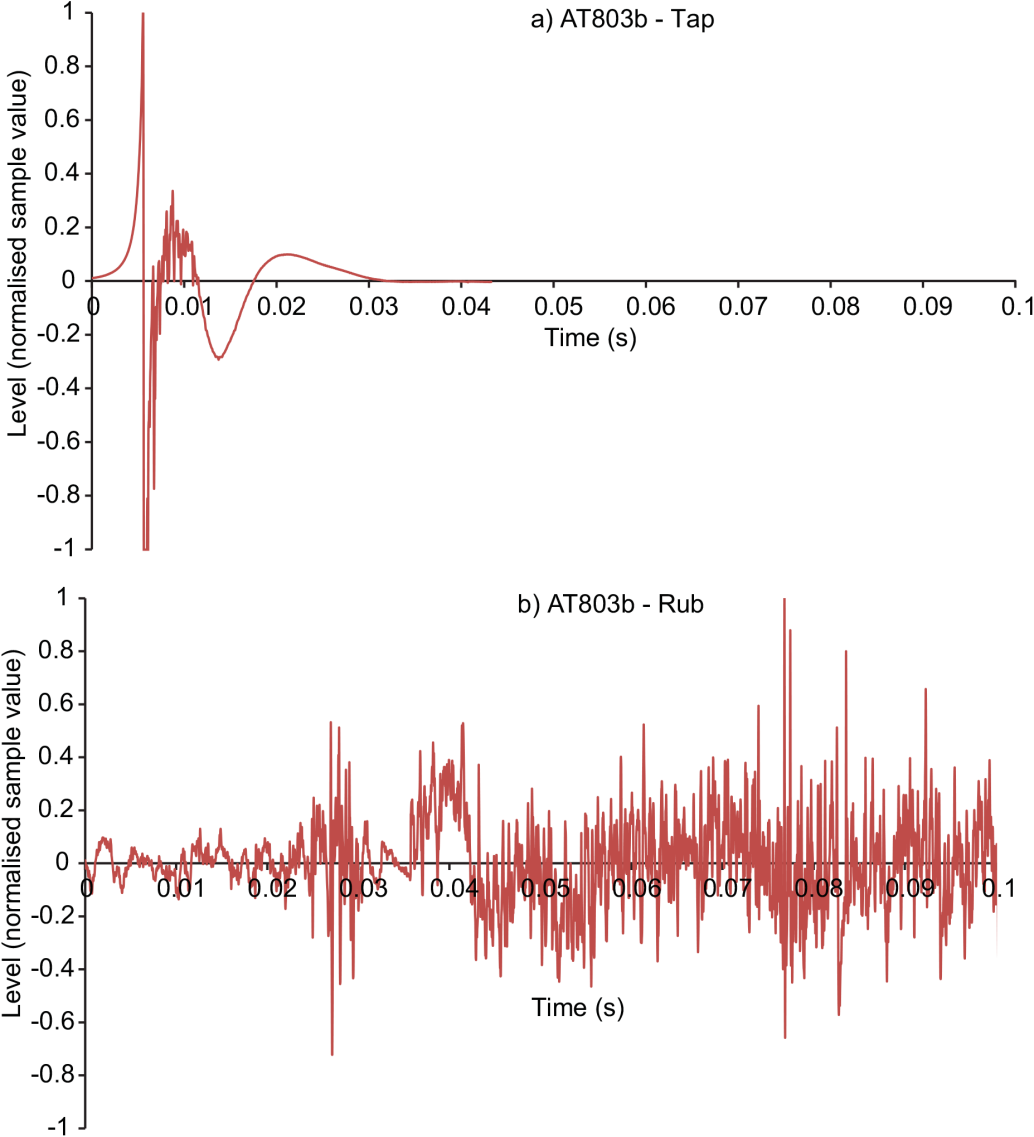
Examples waveforms of microphone handling noises recorded from a dynamic AT803b lapel microphone. a) Tapping and b) rubbing type handling noises.

**Fig 3:**
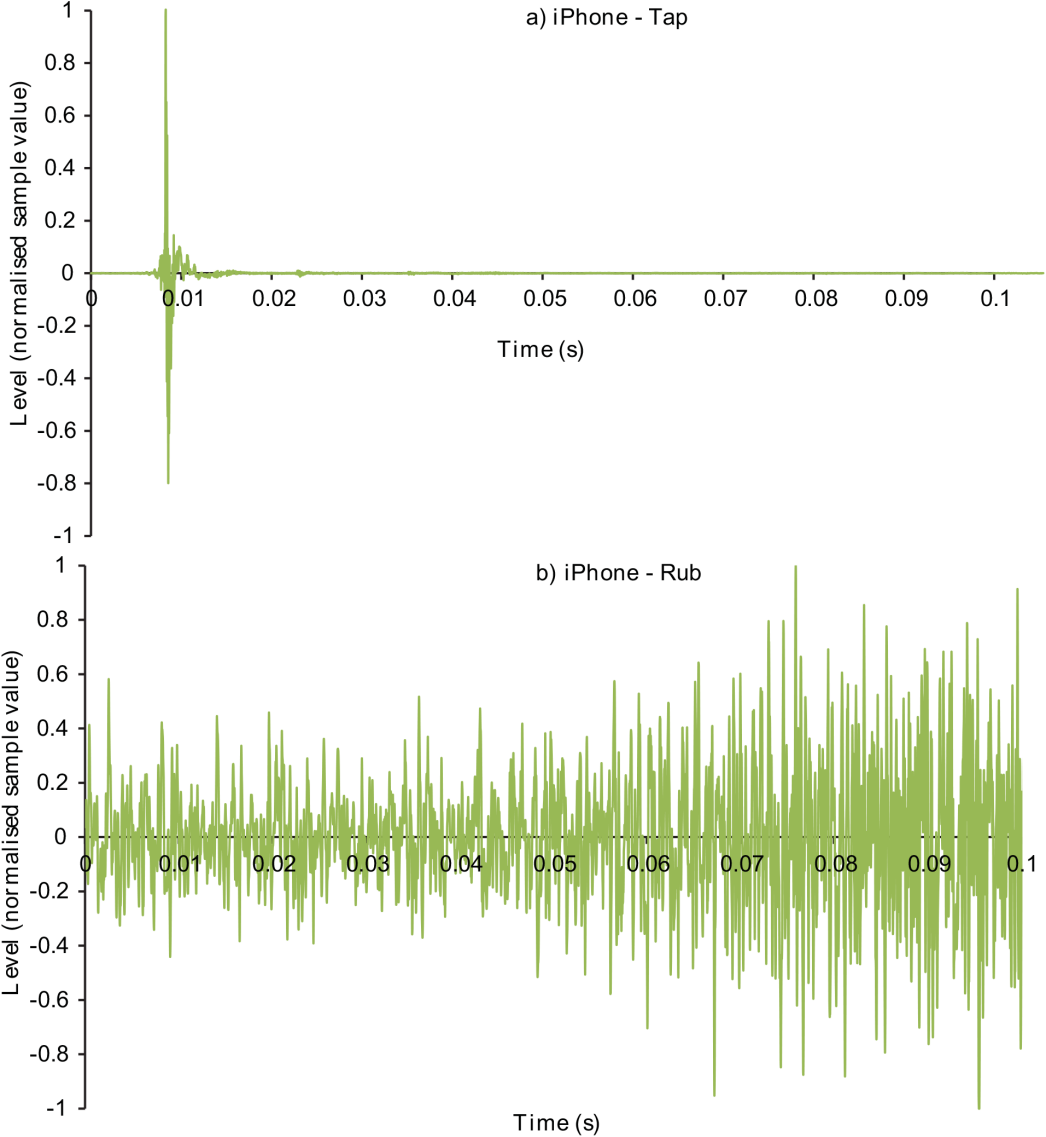
Examples waveforms of microphone handling noises recorded on an iPhone 4. a) Tapping and b) rubbing type handling noises.

**Fig 4:**
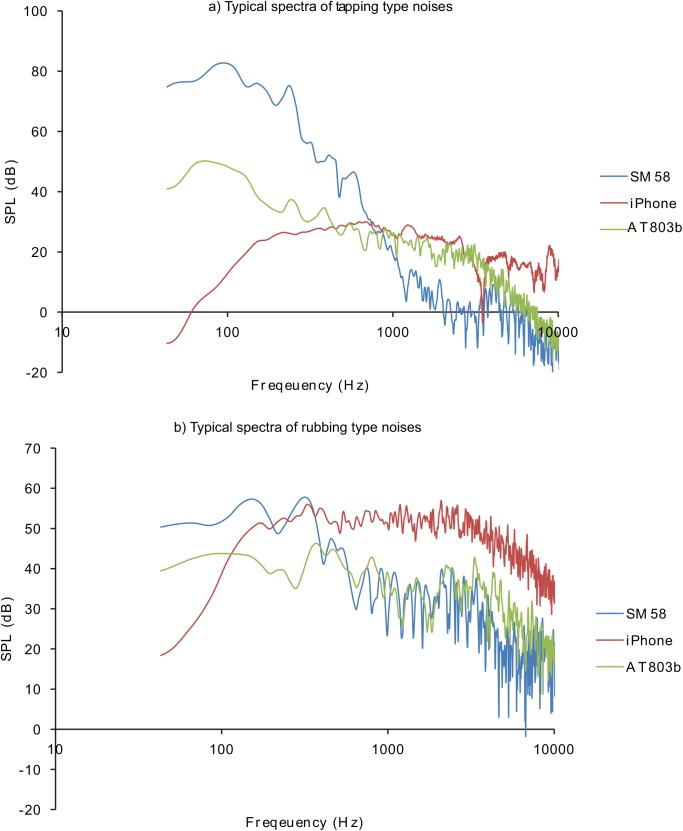
Examples spectra of microphone handling noises. Recorded on the SM58, the AT803b and the iPhone including, a) tapping and b) rubbing type handling noises. The level of the handling noises are calibrated relative to an external sound source.

**Fig 5:**
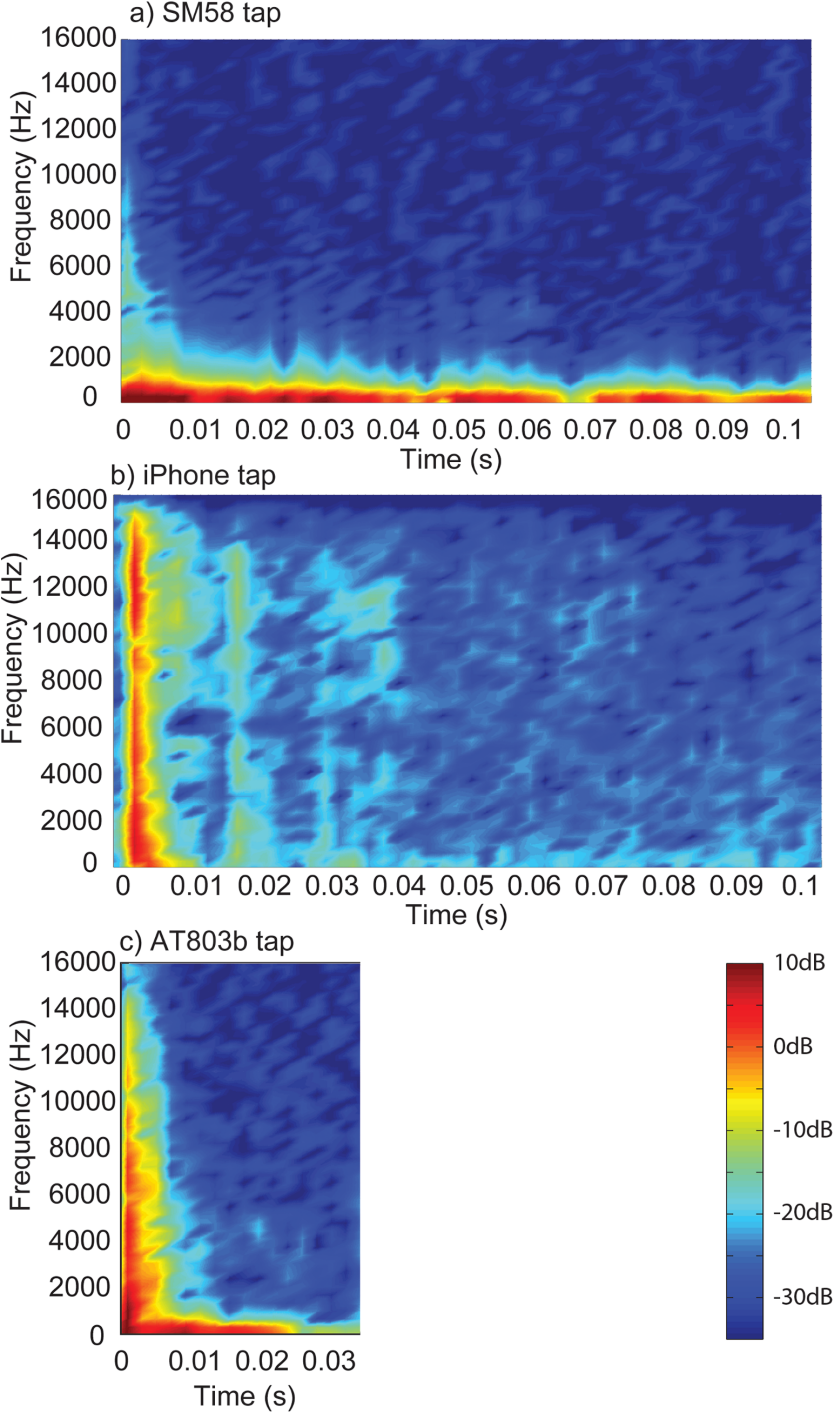
Example spectrograms of tapping type microphone handling noises. Recorded on the a) SM58, the b) iPhone and the c) AT803b.

**Fig 6:**
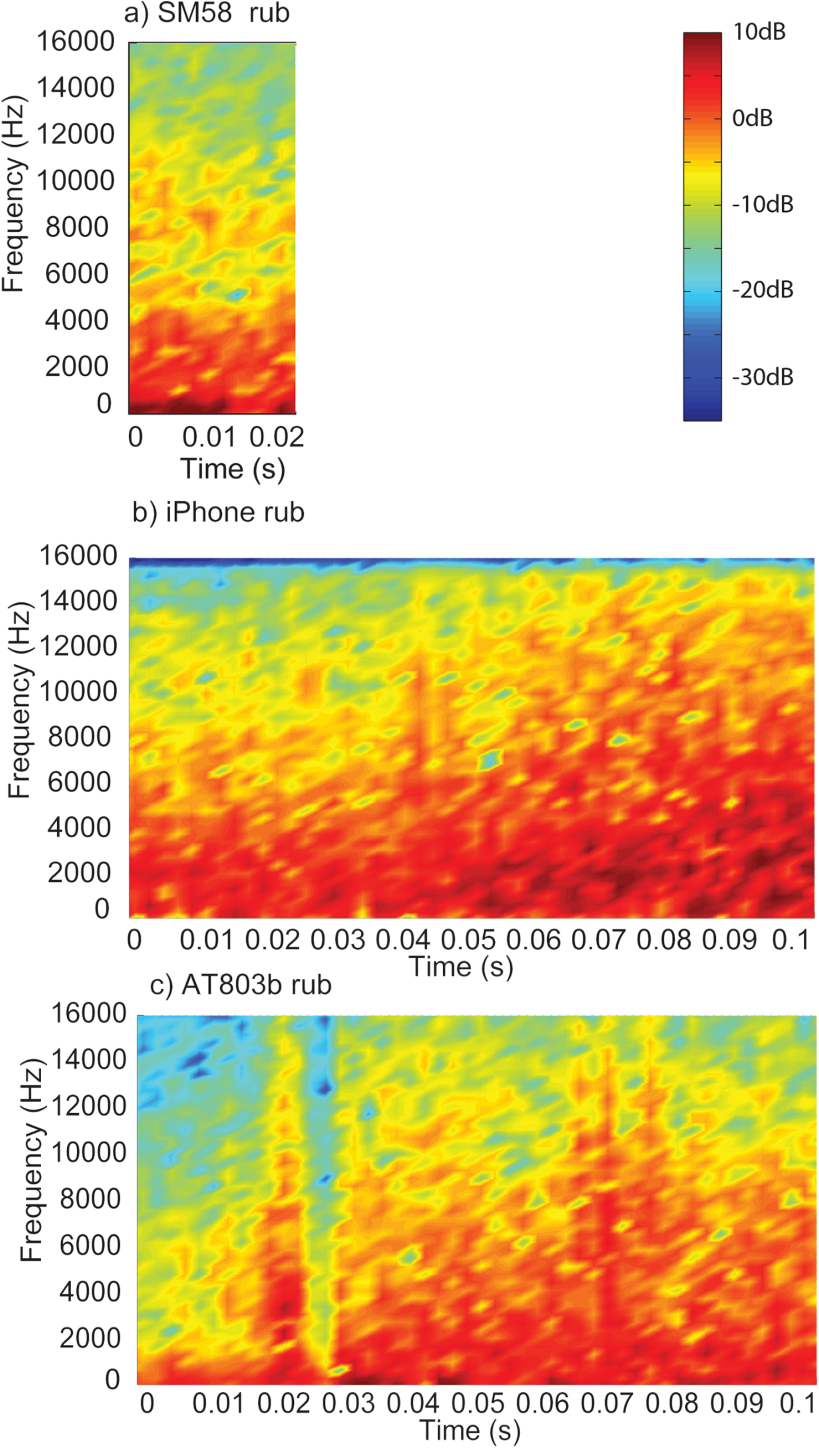
Example spectrograms of rubbing type microphone handling noises. Recorded on the a) SM58, the b) iPhone and the c) AT803b.

Figs 1–3 display the waveforms of six of the handling noise examples for the times selected by the automatic segmentation algorithm. Each waveform is normalised to maximum absolute level and one impact and one rubbing type example for each device are presented. Fig 4 compares the power spectral densities of the selected noises. The SM58 responses show the effects of mechanical resonances indicating the coupling of a number of damped oscillatory systems. The first resonance appears at around 100 Hz, while the second is between 200 and 300 Hz although this varies between sounds. For the AT803b lapel microphone a similar behaviour is found, but with a slightly lower first resonance at around 60 Hz. The temporal response shows that AT803b’s handling noises persist for a shorter time as compared with the SM58. The iPhone impact sounds decay much more quickly than the other two devices, and there is very little energy at lower frequencies due to the presence of a high-pass filter, active by default on the device with a cut-off frequency of about 120 Hz. The spectrum also indicates some resonant behaviour but at a higher frequency of around 1.2 kHz. Examining the response to rubbing excitation shows a noise like broadband response for the AT803b and iPhone devices, while the SM58 retains the resonant behaviour demonstrated by the tapping excitations. Figs 5 and 6 show spectrograms for the same sounds. The waveform level has been normalised to maximum absolute sample value, and 3 ms (128 samples) Hanning windows with 75% overlap were used. The rubbing sounds in Fig 6 show a consistent spectral distribution over time, while the tapping sounds in Fig 5 shows that higher frequencies are attenuated more quickly than lower frequencies.

Table 1 gives summary signal statistics for the handling noises used in the perceptual tests and Table 2 presents data for an additional five commonly used devices. The decay rate was computed using a straight line fit to the Sound Pressure Level (SPL) of the loudest impulsive event within each handling noise. This impulsive event was selected by finding the loudest point in the sound and the point where the sound had decayed by either 40 dB, or to the end of the sound. The dominant spectral peak is the peak frequency with the greatest magnitude. On average, 360 individual handling noises were recorded for each device. The signal statistics were calculated using the MIR toolbox [13], using a window of 1024 samples. Means and 95% confidence limits are presented. The L_Aeq_ of each noise is also presented; this is the equivalent sound pressure level when the device is knocked with respect to a calibrated external sound source.

**Table 1:** Handling noise signal features extracted from the three devices used in the psychoacoustic tests. All signal features were averaged over all handling noises generated for each device. 95% confidence intervals are also presented.

		SM58	AT803B	iPhone 4
**Decay rate (dB/s)**	Rub	8.1 ± 0.7	12.1±1.1	13.2±0.7
	Tap	10.3±0.7	10.3±1.3	15.2±0.8
**Dominant Spectral peak (Hz)**	Rub	103±12	299±95	484±76
	Tap	84.4±6.5	97±59	714±185
**Spectral Centroid (Hz)**	Rub	957±83	2310±236	3146±150
	Tap	637±74	2843±264	4483±126
**Spectral Spread (Hz)**	Rub	2355±107	3025±156	3249±80
	Tap	2047±115	3979±229	3887±49
**L**_**eqA**_ **(dB)**	Rub	88.3±1.1	70.0±1.1	82.3±0.6
	Tap	90.7±1.0	70.1±1.0	66.0±0.9
**Number of Examples**	Rub	222	253	434
	Tap	304	238	361

**Table 2:** Handling noise signal features extracted from an additional five devices. All signal features were averaged over all handling noises generated for each device. 95% confidence limits are also presented.

		ATM25	NT2A	Shotgun Microphone	Sony Camcorder	Dell Laptop
**Decay rate (dB/s)**	Rub	7.3±0.5	8.5±0.5	8.5±0.6	16.4±0.9	17.5±0.6
	Tap	7.8±0.6	11.6±1.0	11.6±1.3	20.8±0.7	18.7±0.6
**Dominant Spectral peak (Hz)**	Rub	84.4±6.1	133±53	133±59	291±47	688±85
	Tap	76.0±6.7	190±102	190±124	351±62	273±53
**Spectral Centroid(Hz)**	Rub	658±39	3376±122	3377±135	2705±171	4255±110
	Tap	470±45	1681±195	1681±239	3673±100	3949±165
**Spectral Spread (Hz)**	Rub	2072±66	4116±66	4116±73	3174±116	4802±90
	Tap	1779±93	2847±143	2847±175	3797±74	4929±127
**L**_**eqA**_ **(dB)**	Rub	85.5±0.7	67.6±0.7	72.3±0.7	60.3±0.8	62.2±0.5
	Tap	94.9±0.9	69.3±1.1	82.5±1.2	60.3±0.8	65.6±0.7
**Number of Examples**	Rub	536	532	434	368	617
	Tap	371	241	161	369	432

### Perceptual Test design

The subjects were presented with a complete podcast (around 3 minutes of audio) from one of three podcasts taken from The Internet Archive [14]: the introduction from a 2013 Building Bridges documentary on Nelson Mandela (stereo, 171 s); an informal discussion of top ten favourite actors taken from the 3 Guys in a Bedroom Talking About Movies Podcast from 2011 (stereo, 180 s), and a section of the original broadcast of the 1938 radio play War of the Worlds (mono, 187 s). These were chosen to represent three types of radio formats: documentary, informal chat and drama.

At six (non-predictable) points during playback, the audio was paused and a true or false question relating to the content of the podcast was asked. The purpose of this question was twofold, first to focus the subject’s attention on the source material, and second to provide a measure of engagement with the podcast. The questions were simple and straightforward to answer for anyone concentrating on what people were saying in the podcast. On average these questions appeared every 30 seconds, with the shortest time between questions being 20 seconds and the longest 40 seconds.

Handling noises were added to the podcasts using the following rules. For each podcast presentation the microphone type was kept constant and 15 noises were presented at every permutation of five levels and three lengths. Noises were superimposed over the whole of the podcast with a spacing between noises chosen randomly between 5 and 10 seconds. Noises within 2½ seconds of a question were not added. 5 levels of handling noises were used set by the peak signal to noise ratio of -20, -10, 0, 10 and 20 dB. The peak level of the podcasts was computed as the average of the peak level over one second, 50% overlapping windows. Rubbing and tapping sounds were included in each presentation. Each noise sequence was generated separately for each podcast (due to the differing location of the questions). And for each podcast and microphone type there were 4 different random noise sequences available.

Separate audio files where generated for every combination of 3 podcasts, 6 question sections, 3 microphones and 4 sequences (216). Mono sounds were all converted to stereo using equal levels for each channel. Files were encoded as stereo 128 kbits/s, using MP3 or OGG encoding; which codec was replayed depended on the browser that the test subject was using. To remove ordering effects, presentation orders were randomised in terms of podcast, microphone type and handling noise sequence.

Subjects taking the test were self-selecting. The experiment was publicised via social media. On the first screen subjects were asked for their consent. The second screen included a short clip of audio for subjects to check that the sound was working on their computer. Next, subjects were asked for some contextual data: (1) The reproduction equipment (headphones, laptop/tablet/mobile/internal loudspeakers, external loudspeakers or other/don’t know). (2) The background noise of the listener’s environment (very quiet, quiet, noisy, very noisy) and (3) Age (in decade ranges).

The detailed instructions were on the next screen, ‘Audio recordings found on the internet are of mixed quality. Some can be very good and contain no recording errors. Others contain noises that the person making the recording did not intend to be there. This experiment requires you to listen to a short section of a podcast or radio show and answer any questions about the clip that appear on your screen. Occasionally you might also hear unwanted noises or errors in the clip. If you think you hear an unwanted sound in the recording click the *Pause* button immediately. You will be then asked how much the unwanted sound affected the overall audio quality at that point in the recording.’

The next screen presented the podcast. All the audio was heard once and could not be replayed. When the participants pressed the pause button because they had heard an unwanted sound, a question then appeared asking them how much the unwanted sound affected their perception of the overall audio quality at that point. This was rated on a five point ITU degradation scale [15] (not at all, not very much, a little bit, quite a lot, a lot). A button allowed subjects to return to the podcast without rating.

#### Ethics

The College of Science and Technology’s Research Ethics Panel at the University of Salford has delegated powers to make judgements regarding ethical approval of research studies on behalf of the Academic Audit and Governance Committee of the University. The panel granted ethical approval for the experiment reported in this paper (reference CST 11/19) including approving the consent process. On the first page of the website, a description of the experiment and a consent statement was given. The instruction was, ‘Please read the following statement and click *Take Part Now!* if you agree to participate.’ It is assumed that by clicking the button participants had given consent. The anonymised, raw data from the experiment can be downloaded from S1 Dataset.

## Results

### Participant summary

1206 participants took part in the study, of whom 629 completed at least one whole podcast. The median age range was 20–29 years. Participants who did not listen to at least one complete podcast or who did not engage with the task (i.e. those who did not identify at least one instance of unwanted noise in the audio) were removed from the final sample, leaving a total of 585 sets of ratings for analysis. Dropout rates in internet based tasks vary considerably [16], particularly in the absence of financial incentive to participate, but retention in this work compares well to previous Web experiments using speech stimuli [17]. Of those in the final sample, 54.9% listened on headphones, 29.9% on laptop/tablet/mobile/internal loudspeakers, 14.2% on external loudspeaker, and 1% on other/don’t know. 24.1% reported the listening environment as very quiet, 63.2% quiet, 12% noisy and 0.7% very noisy. If a subject did not press the button to rate the quality after a sample contained handling noise, this was automatically assigned a degradation rating of ‘not at all’.

### Predicting quality scores

An ordinal regression model using a logit link function and proportional cumulative odds was used for this part of the analysis. The regression model is therefore [18]:

$$ln\left(\frac{p_{j}}{1-p_{j}}\right)=\alpha_{j}+\beta \cdot SNR+\varepsilon_{k}C_{k}$$ (1)

Where *p*_*j*_ is the probability that the degradation was rated in category *j* or below, *α*_*j*_, *β and ε*_*k*_ are coefficients that are adjusted to fit the model to the data, *SNR* is a signal to noise ratio, and *C*_*k*_ a nominal contextual variable (e.g. microphone type) with subscript *k* indicating category number.

Initial analysis showed that signal to noise ratio was the strongest significant predictor for the cumulative probabilities. Signal to noise ratios can be drawn up in different ways, however, and so it was necessary to investigate which correlated best with the subjective ratings. A number of variations of signal to noise ratios were compared including: the ratio of the average level of the signal and noise (L_eq_ based SNR); the ratio between the signal and noise L_10_ levels (L_10_ is a percentile measure of the level exceeded for 10% of the time and is often used to quantify impulsive sound levels); the peak signal to noise ratio and variations using A-weighting and also SNR parameters where the signal level was integrated over the whole file rather than just over the time when the particular noise was present.

To test which signal to noise ratio parameter was the best predictor of the degradation scores, an ordinal regression analysis was done for each parameter and the best model selected as monitored via the pseudo-R^2^ value. Other contextual variables were not used (i.e. *C*_*k*_ = *ε*_*k*_ = 0).

The A-weighted signal to noise ratio, where the signal level is calculated over the whole signal, correlated best with opinions of quality. (Note, two other forms of determining signal to noise ratios that used a masking model accounted for a similar amount of variance, but were rejected as being more complex). This suggests that the perceived influence of the handling noise on quality is not related to the instantaneous signal to noise ratio, but to how the level of each noise relates to a subject’s memory of the average level of the podcast.

**Fig 7:**
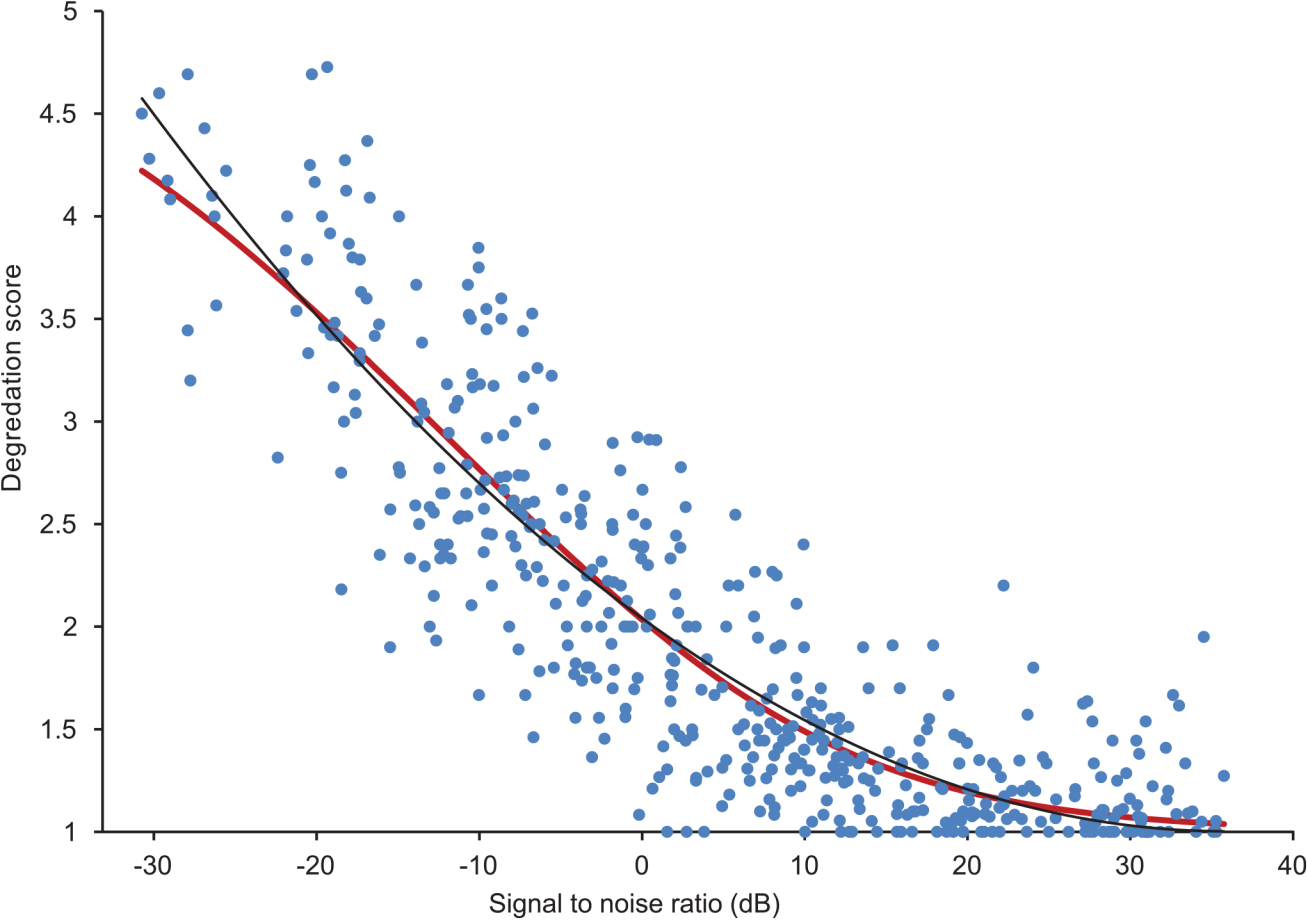
Mean opinion score on the degradation scale vs signal to noise ratio. Dots: average for each sample. Red line: estimation using ordinal regression model. Black line: polynomial fit to the ordinal regression model.

Fig 7 shows the mean opinion scores for each sample versus the most perceptually relevant signal to noise ratio calculation. The mean opinion scores were calculated by treating the degradation scores as interval data and averaging the values across subjects. The figure also shows an estimate from the regression model, which was calculated as follows. For each signal to noise ratio, the output from Eq 1 was used to estimate the probability distribution of the expected scores on the 5-point degradation scale. The mean opinion score was then the mean calculated from the probability distribution.

The ordinal regression model had a Cox and Snell pseudo-R^2^ [19] of 0.385. 59% of predictions from the model gave the right value on the degradation scale and 21% were wrong by 1 position on the scale. For an experiment that was carried out across the Internet, with all the additional variability that this introduces into the scores, this is a reasonably large amount of variance to be able to model. The log likelihood showed the fitted model to be significantly better than the intercept-only model. Testing the assumption of proportional odds indicates there was a significant chance (p = 0.001) that the coefficient *β* was not the same for all categories *j*. It is known, however, that this test of parallel lines often results in rejection of the proportional odds assumption [20]. As Fig 7 shows, the ordinal regression allowed a reasonable prediction of the mean opinion score, and so the analysis continued to use this model. The model parameters were: α_1_ = -0.185, α_2_ = 0.736, α_3_ = 1.929, α_4_ = 3.248, α_5_ = 0; β = 0.108. (Note, the value of β is small, but in Eq 1 it is multiplied by the SNR that is numerically large).

It would be useful to have a simple equation to predict mean opinion scores, whereas the ordinal regression outputs cumulative odds. A quadratic best fit line was calculated on the output of the regression model shown in Fig 7. This yielded the following equation for the degradation, *D* on a continuous scale between 1 and 5:

$$D=2.04-5.78\times 10^{-2}\;SNR+0.803\times 10^{-4}\;SNR^{2}$$ (2)

#### Influence of other factors

The experiment also gathered other data that might explain some of the remaining variance in degradation scores. The general analysis method was to add the contextual data into the ordinal regression and to monitor whether it significantly improved the model in terms of the pseudo-R^2^ and number of correct predictions.

A number of the contextual variables did not improve the model. There were two methods for generating microphone noise, one used a tapping motion, the other rubbing, but adding the noise type as a contextual variable did not improve the model. The subjects were asked to report how noisy their listening environment was. When this was added to the model—excluding the small number of ‘very noisy’ replies–but this contextual variable did not improve the model.

Three other contextual variables account for some more of the variance, but their effects were small. For the microphone types, the model parameters were: ε_m1_ = -0.3±0.1 (Shure mic, p<0.001), ε_m2_ = 0.2±0.1 (iPhone, p<0.001) and ε_m3_ = 0 (AT mic); pseudo-R^2^ = 0.399 and 59% predictions correct. This means that for a given SNR, handling noise produced by the iPhone was most degrading to quality, followed by the AT mic and lastly the Shure microphone degraded the quality least. For the three podcasts, the model parameters were: ε_p1_ = -0.4±0.1 (Mandela, p<0.001), ε_p2_ = -0.5±0.1 (Top Ten Actors, p<0.001) and ε_p3_ = 0 (War of the Worlds); pseudo R^2^ = 0.389 and 60% predictions correct. This means that the War of the Worlds podcast was more degraded by handling noise than the other two. A difference might have been anticipated because the War of the Worlds audio stems from a very old recording with narrow bandwidth and had at some point in the past been recorded from vinyl and so suffered from the typical clicks and pops. For the reproduction equipment, the model parameters were: ε_r1_ = 0.2±0.1 (headphones, p<0.001), ε_r2_ = -0.6±0.1 (laptop/tablet/mobile/internal loudspeakers, p<0.001), ε_r3_ = 0 (external loudspeakers); pseudo R^2^ = 0.399 and 60% predictions correct. Built-in loudspeakers tended to degrade quality less than external loudspeakers whereas headphones tended to degrade quality more. (When testing the reproduction equipment, the ‘other’ answers were not considered because of the small number of responses in that category.)

The experimental method was designed to explore the effect of listening mode on quality scores. Listeners were asked questions about the podcast, and whether they got this right or wrong is assumed to be a measure of how much attention subjects were paying to the foreground sound. 63.4% of the questions were answered correctly. However, adding the percentage of questions each subject answered correctly as a variable in the ordinal regression model produced no statistically significant improvement to the model.

### Absolute threshold of detection of handling noises

A binary logistic regression was used to model the detection of handling noise. The dependent variable was whether or not a handling noise was audible, thus a binary outcome. Detection was defined as whether the button was pressed within three seconds of the start of the noise. Three seconds was chosen as this was the length of the longest handling noise used in the test. The model uses the SNR to predict whether handling noises are detected. The Box-Tidwell method was used to confirm the statistical validity of the model [21]. The statistical validity test was significant (χ^2^ = 3241, df = 1, p<0.001, Cox and Snell R^2^ = 0.309). The model correctly predicts 76% of cases (N = 8775).

The model provides the odds ratio of a subject pressing the key because they heard handling noise, to them missing the noise, as:

$$\log\left(\frac{p}{1-p}\right)=0.404-0.097SNR$$ (3)

The threshold at which 50% of subjects identify a handling noise was 4.2 ± 0.6 dBA.

The tests also measured the false positive rate. On average, subjects reported a noise that was not attributable to microphone handling noise about every 26 seconds, with no significant difference between podcasts.

## Discussions and Conclusions

A psychoacoustic experiment was carried out to quantify how microphone handling noise affects the perception of audio quality. The experiment used a variety of measured handling noises that were then added to three speech podcasts. A representative experimental design was developed to encourage listening to the foreground speech rather than subjects analytically listening for degradations. Tests were carried out over the Internet, so degradation scores were representative of everyday listening equipment and environments, and so the opinion of naive rather than expert listeners could be obtained.

A model was developed that allows the mean opinion score for quality degradation on a five point scale to be predicted from the signal to noise ratio of the handling noise. It was found that the most perceptually relevant measure for signal to noise ratio was that based on the A-weighted signal to noise ratio, where the signal level is calculated over the whole signal. Using a logistic regression model, the threshold at which 50% of subjects detected handling noise in the programme material was found to be 4.2 ± 0.6 dBA. An analysis of the influence of other factors revealed small or insignificant effects.

To examine how the intermediate questions that were designed to focus listener’s attention on the foreground sound altered the ratings, the experiment was re-run with the questions removed. The threshold at which 50% of subjects detected handling noise in the programme material increased to 7.0 ± 0.9 dBA, showing that answering the questions engaged attention resources and therefore affected the rating of quality. There was no significant difference in drop-out rates between the two experiments, χ^2^ (1, *N* = 2035) = .869, *p* = .35, indicating that asking questions did not improve engagement with the test.

The War of the Worlds podcast led to significantly higher degradation ratings for the added handling noises. This indicated a higher sensitivity of subjects to the handling noise cases when presented over this podcast. Because this older podcast has a much narrower frequency bandwidth, the absence of frequency masking might have left the added handling noise more exposed, thus explaining the higher degradation attributed to them. This is an interesting implication, but given that only three podcasts were investigated, further investigation is needed to draw more definite conclusions.

The next stage for this work will be to develop a signal processing algorithm to detect handling noise present in recordings. The perceptual results presented in this paper will be used to construct appropriate scales for the output of the machine learning algorithms.

## Supporting Information

S1 Audio Microphone handling noise database. (ZIP)

S1 Dataset Subject responses. (ZIP)

## References

[1] Jackson I, The Good Recording Project Blog, What you told us about recording audio: an overview of our web survey [Internet], Salford: Iain Jackson 2012 - [cited 2012 Nov 20]. Available: http://www.goodrecording.net/211/.

[2] Shure Customer Help [Internet], Handling Noise of Microphones. 2003 - [cited 2015 Jan 20]. Available: http://shure.custhelp.com/app/answers/detail/a_id/2957/~/handling-noise-of-microphones.

[3] ITU-R (2003) Method for the subjective assessment of intermediate quality levels of coding systems. ITU-R Recommendation BS.1534, International Telecommunications Union, Geneva, Switzerland.

[4] ITU-R (2015) Methods for the subjective assessment of small impairments in audio systems. ITU-R Recommendation BS.1116, International Telecommunications Union, Geneva, Switzerland.

[5] ITU-T (1998) Subjective audiovisual quality assessment methods for multimedia applications. ITU-T Recommendation P.911, International Telecommunications Union, Geneva, Switzerland.

[6] Traux B, Acoustic Communication Volume 1, 2nd ed. GreenWood Publishing Group; 2000 p. 163–165.

[7] Brunswik E, Perception and the Representative Design of Psychological Experiments. University of California Press; 1956 p. 154.

[8] Araujo D, Davids K, and Passos P, Ecological validity, representative design, and correspondence between experimental task constraints and behavioral setting: Comment on Rogers, Kadar, and Costall. Ecol. Psychol. 2007;19: 69–78.

[9] Berglund B, and Nilsson ME, On a Tool for Measuring Soundscape Quality in Urban Residential Areas. Acta Acustica uw Acust. 2006;92: 938–944.

[10] Jackson IR, Kendrick P, Cox TJ, Fazenda BM, Li FF, Perception and automatic detection of wind-induced microphone noise. J Acoust Soc Am. 2014; 136: 1176–1186. doi: 10.1121/1.4892772 25190392 10.1121/1.4892772

[11] Västfjäll D. The “end effect” in retrospective sound quality evaluation. Acoustical Science and Technology. 2004 25: 170–172.

[12] Electroacoustics—Sound level meters—Part 1: Specifications, International Electrotechnical Commission (IEC), IEC 61672–1, May 2002.

[13] Lartillot O, Toiviainen P, A Matlab Toolbox for Musical Feature Extraction From Audio, International Conference on Digital Audio Effects, Bordeaux; 2007.

[14] The Internet Archive. [Online]. Available: https://archive.org/. [Accessed: 15-Dec-2014]

[15] Methods for subjective determination of transmission quality, Int. Telecommun. Union, Geneva, Rec. ITU-T P.800, 1996.

[16] Musch J, and Reips UD. (2000). A Brief History of Web Experimenting In Birnbaum M. H. editors. Psychological experiments on the internet, San Diego: CA Academic Press; 2000. pp. 61–87.

[17] Knoll MA, Uther M, and Costall A. Using the Internet for speech research: an evaluative study examining affect in speech. Behaviour & Information Technology, 2011 30(6), 845–851.

[18] O'Connell AA, Logistic regression models for ordinal response variables, SAGE Publications; 2006 p. 31.

[19] O'Connell AA, Logistic regression models for ordinal response variables, SAGE Publications; 2006 p.21

[20] O'Connell AA, Logistic regression models for ordinal response variables, SAGE Publications; 2006 p.29

[21] Binomial Logistic Regression using SPSS, [Internet], Laerd Statistics, 2013 - [cited 2015 Jan 1] Available: http://statistics.laerd.com/spss-tutorials/binomial-logistic-regression-using-spss-statistics.php.

