# Supplementary material for: Microphone Handling Noise: Measurements of Perceptual Threshold and Effects on Audio Quality
Source: PLoS One. 2015 Oct 16;10(10):e0140256. doi: 10.1371/journal.pone.0140256 (PMC4608586; doi:10.1371/journal.pone.0140256)
Supplement: S1 Audio — (ZIP) [file pone.0140256.s001.zip › S1 Audio readme.rtf]

Microphone handling noise database – description

This zip archive contains microphone handling noises recorded on 8 different devices.  For each device 2x4 minutes of handling noises were recorded in a semi-anechoic chamber on 14th January 2014 at the University of Salford.  Two types of handling noises were recorded for each device:

Tapping:  These noises were generated by operating the device in such a manner to produce impulsive sounds, such as tapping of the device by the operators finger, and against other objects.

Rubbing: These noises were generated by rubbing the device against either the operators hand, clothing or other soft material to create a more sustained noise like excitation.

Each file has a 1 kHz calibration tone at the start, played back over a loudspeaker @1m in anechoic conditions with the level set so that at the device's position there will be a SPL of 84 dB Linear unweighted. The equivalent SPL of each recorded noise can be extracted by multiplying the wav sample level, which is bounded between -1 and 1 by:

	i		MIC				Rub			Tap
    1 		ATM25			   	1.3998e+06  	1.5461e+06
    2 		NT2A				4.2072e+05 	1.8727e+05
    3 		SM58				1.7826e+06   	1.7457e+06
    4 		Shotgun			9.9545e+05   	1.0591e+06
    5 		Sonycam			8.2352e+04   	8.2573e+04
    6 		at803b			5.5009e+05   	5.4376e+05
    7 		iPhone			1.1868e+05   	1.0382e+05
    8 		laptopmic-dell		8.5989e+04   	8.6807e+04

The audio has been compressed using a lossless codec.
