# Supplementary material for: Microphone Handling Noise: Measurements of Perceptual Threshold and Effects on Audio Quality
Source: PLoS One. 2015 Oct 16;10(10):e0140256. doi: 10.1371/journal.pone.0140256 (PMC4608586; doi:10.1371/journal.pone.0140256)
Supplement: S1 Dataset — (ZIP) [file pone.0140256.s002.zip › S3 Dataset readme.rtf]

This zip archive is a csv file containing the results from the perceptual study, a total of 42657 rows, where each row contains either:

·	A response were a noise was present on the podcast and the subject pressed the button
·	A response where a noise was not present in the podcast and the subject pressed the button
·	No response were a noise was present
·	No response where no noise was present on the podcast 

In all cases the response represents a 3 second section of audio, the variables in the columns are:

HitTrue	handling noise present =1 (0 no noise)	
HitPerson	Person pressed button =1 (0 no press)	
True person ID	number representing a unique ID for a person	
Qual	If  button pressed, user make degradation rating (0-4), 0 is written to this column if not pressed	
age	(0)0-9,(1)10-19(2)20-29(3)30-39(4)40-49(5)50-59(6)60-69(7)70-79(8)80+	
mic	(1)SM58(2)iPhone(3)at803b	
rubtap	(0)rub(1)tap	
questionCorrect	Was the question regarding the podcast content  that ends this section correct? (0 or 1)	
podcast	(1)Documentary(2)Actors podcast(3)war of the worlds	
loc	(0)very quiet(1)quiet(2)noisy(3)very noisy	
repro	(0)headphones(1)laptop/tablet/mobile internal loudspeakers(2)External LoudSpeakers(3)Other / Don't know	
level_A	Peak SNR used to set level of signal	
length	(1)short(2)medium(3)long	
SNRA_global	A weighted SNR, signal level computed over whole podcast, noise over length of noise.	
Qright	out of 6 questions about content correct per subject	
NoiseNoticed	out of 15 noises noticed per subject	
